# ClimMob: Software to support experimental citizen science in agriculture

**DOI:** 10.1016/j.compag.2023.108539

**Published:** 2024-02

**Authors:** Carlos Quirós, Kauê de Sousa, Jonathan Steinke, Brandon Madriz, Marie-Angélique Laporte, Elizabeth Arnaud, Rhys Manners, Berta Ortiz-Crespo, Anna Müller, Jacob van Etten

**Affiliations:** a Digital Inclusion, Bioversity International, Montpellier, France; b Department of Agricultural Sciences, Inland Norway University of Applied Sciences, Hamar, Norway; c MrBot Software Solutions, Cartago, Costa Rica; d International Institute of Tropical Agriculture, Kigali, Rwanda

**Keywords:** Crowdsourcing, Decentralized experiments, Digital inclusion, Tricot approach

## Abstract

Experimental citizen science offers new ways to organize on-farm testing of crop varieties and other agronomic options. Its implementation at scale requires software that streamlines the process of experimental design, data collection and analysis, so that different organizations can support trials. This article considers ClimMob software developed to facilitate implementing experimental citizen science in agriculture. We describe the software design process, including our initial design choices, the architecture and functionality of ClimMob, and the methodology used for incorporating user feedback. Initial design choices were guided by the need to shape a workflow that is feasible for farmers and relevant for farmers, breeders and other decision-makers. Workflow and software concepts were developed concurrently. The resulting approach supported by ClimMob is triadic comparisons of technology options (tricot), which allows farmers to make simple comparisons between crop varieties or other agricultural technologies tested on farms. The software was built using Component-Based Software Engineering (CBSE), to allow for a flexible, modular design of software that is easy to maintain. Source is open-source and built on existing components that generally have a broad user community, to ensure their continuity in the future. Key components include Open Data Kit, ODK Tools, PyUtilib Component Architecture. The design of experiments and data analysis is done through R packages, which are all available on CRAN. Constant user feedback and short communication lines between the development teams and users was crucial in the development process. Development will continue to further improve user experience, expand data collection methods and media channels, ensure integration with other systems, and to further improve the support for data-driven decision-making.

## Highlights

• ClimMob is a software package that facilitates experimental citizen science in agriculture.
• ClimMob was designed to support tricot, a citizen science approach which allows farmers to make simple comparisons between crop varieties.
• ClimMob facilitates implementation of experimental citizen science at scale.

## 1 Introduction

“Crowdsourced citizen science” is a form of participatory research that has recently been introduced to the agricultural sciences (Minet et al., 2017; Ryan et al., 2018). The agricultural sciences are eminently experimental in nature, and experimental citizen science offers new ways to organize on-farm testing of crop varieties and other agronomic options to inform breeding programs on the performance of breeding products, or to inform advisors who generate recommendations for farmers. Experimentation in agriculture can also involve tests in other use contexts (processing plants, markets, homes), with different types of participants, including farmers, processors, and consumers. Compared to other styles of participatory research, citizen science can reduce the costs and effort per data point, enhance participant engagement, facilitate the management of participatory trials, and produce insights that are difficult to obtain with other styles of participatory research (van de Gevel et al., 2020). Specifically, insights on how varieties and agronomic options interact with different environmental conditions are difficult to obtain from small, highly localized trials. To reach scale, digital support is an important ingredient of most citizen-science initiatives as it facilitates data standardization, exchange, aggregation, and participant recruitment, and reduces the cost of communication.

This article describes ClimMob, a software package that facilitates experimental citizen science in agriculture, specifically designed to support “triadic comparisons of technology options” (tricot), a citizen science approach (van Etten, 2011, Steinke et al., 2017, van Etten et al., 2019a, van Etten et al., 2019b). This specific application of citizen science required specialized software to enable streamlined support of the full experimental cycle, from experimental design to report creation. This paper focuses on the software development only, as the scientific aspects of the methodology have been described elsewhere (van Etten et al., 2019a, van Etten et al., 2019b). The platform has been used in a series of recently published studies (Moyo et al., 2021, de Sousa et al., 2021a, de Sousa et al., 2021b, Brown et al., 2022, Olaosebikan et al., 2023). An updated review of methodological progress is forthcoming in a separate paper.

In the following sections, we describe the software design process, including our initial design choices, and subsequent iterations to add more features and address user feedback. Then, an overview of the architecture and functionality is provided. We also report on a survey to assess the user experience with ClimMob. We conclude the article by providing a self-reflection of the process and a short prospect for future development of the software.

## 2 Design process

**Fig. 1:**
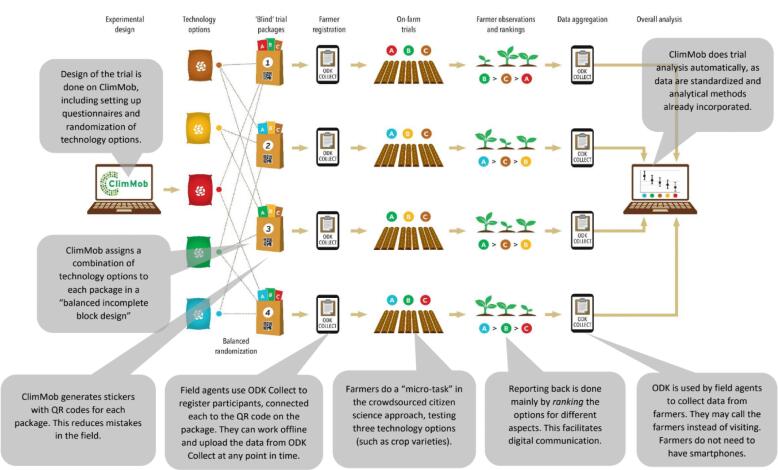
Overview of the tricot approach.

The development of ClimMob took place as part of the overall methodology development of the triadic comparisons of technology options (tricot) approach (van Etten et al., 2019a). This approach was inspired by crowdsourced citizen science, which distributes a large number of small tasks to a large number of participants and combines the resulting data for analysis (Hand, 2010, Hochachka et al., 2012). In tricot, each participant receives a package containing a combination of three technology options to evaluate from a pool of several options (which corresponds to an “incomplete block” experimental design). In much of the initial work, these technology options were crop varieties, but other technology options can also be evaluated. At present, the approach has also been applied to fertilizers and food products derived from different varieties. Participants do not know the identity of the technology options, as trials are blind. In the case of farming technologies, a farmer takes the package home, plants the seeds, and does observations independently, evaluating the technology options by ranking each for different characteristics, aspects of performance, and preferences. Additional quantitative measurements are also possible. Data is then aggregated and analyzed using an automated workflow for quick feedback (∼10 min, depending on the size of the dataset). Fig. 1 provides an overview of the tricot approach.

Very early in the design process, we realized that it was necessary to digitally support *all* the steps of the experimentation cycle. This implied that we needed to make a series of design choices related to both the experimental approach and the digital software to support it (van Etten, 2011, van Etten et al., 2019a):

● *Standardize the experimental design to a large extent*. This minimalistic focus was intended for researchers to easily design and run experiments using the digital platform without much external help.
● *Simplify data collection formats*. Participants share their observations by ranking three technological options (from best to worst, or highest to lowest, etc.). Ranking is easy, requires little training, and leads to fewer errors than rating, which requires calibration to be consistent (Halekoh and Kristensen, 2008). Ranking also facilitates data collection through digital mobile technologies. Quantitative measurements can be accommodated, but are not the main or default method for data collection.
● *Use open-source software, reuse/repurpose existing code or implement new code in a modular way*. This modular design makes it easy to maintain code, add new modules, or use existing modules independently and in different combinations.

**Fig. 2:**
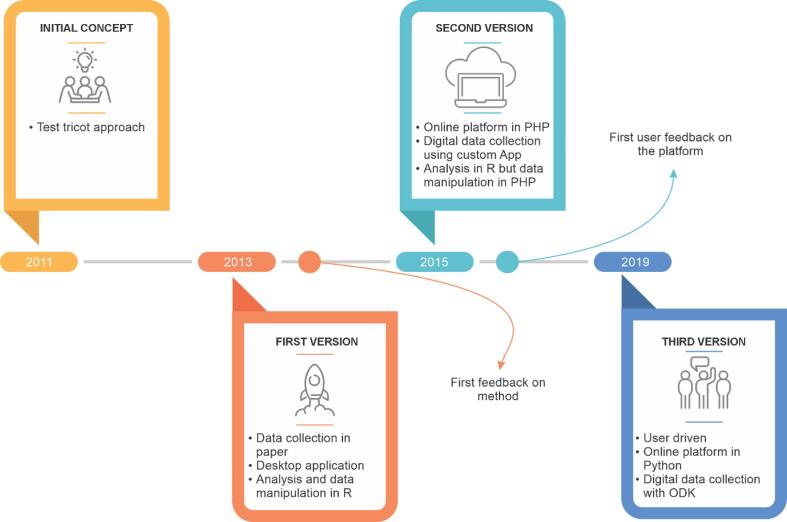
Summary timeline of the development of ClimMob.

The design process was done in several stages, from a quick prototype implementation to the integration of the different concepts, to the current fully-fledged platform that is being used by a large user base. See Fig. 2 for a summary timeline. The first version of ClimMob (2013) was written by co-author JvE in R, a programming language and environment (R Core Team, 2019). An important reason for selecting R was that the selected analytical procedures were available in this language (originally, it relied on Turner and Firth, 2012). Also, the R language allows constructing dialogue windows, which made it easy to create an initial prototype (based on the strategy described in Lawrence and Verzani, 2012). Although this prototype could only be run offline and was not published, it was used in a small number of pilot projects. The package supported the trial design and automatic report generation, but not the data collection. The pilot projects generated various lessons on the feasibility of the approach and issues of communication. These projects revealed the challenges in organizing the data collection in a standardized way. Collaborators used Microsoft Excel to digitize data collected on paper forms. This often resulted in missing data or the need for excessive data cleaning efforts. Another issue was collaboration across distance and over time, sometimes further complicated by the high turnover of field staff. The tricot approach required intensive accompaniment to avoid projects falling back on previous standard routines or behaviors, such as data collection on paper, delayed reporting, or ill-advised modifications of the procedures during trial execution. It was clear that digital support for the entire cycle was needed, including electronic data collection, and providing online guidance for all steps. It was thought that by organizing the trial process from beginning to end in a streamlined, digital way, users would also be gently discouraged from falling back on previous standard routines in the absence of hands-on guidance.

The prototype in R facilitated the creation of a second version in 2015, as the basic concepts could now be easily communicated to a team of software developers. This second version separated the R analysis code from the interface and included features for data collection and data cleaning. It was implemented as an online platform, programmed in the PHP language. It helped users to manage a whole trial cycle and the resulting data. An Android app was developed using Adobe PhoneGap to enable the collection of data using mobile devices and send the submissions to the online platform. This second version of ClimMob established the main concepts and elements of an integrated platform with mobile data collection in a usable tool that allowed to gain feedback from a first group of users, who were collaborators in projects led by Bioversity International. User feedback led to many improvements in the software. One important way in which feedback was collected was through close observation of participants in courses on tricot and the use of ClimMob. Also, regular conversations with users were important.

As the user base increased, we realized that a new version of ClimMob was needed that would be more extensible, flexible, modular, and standardized. For example, users indicated their need to collect different types of data. Also, a need for data standardization was clear as different trials named traits in slightly different ways, precluding easy data aggregation across seasons and across different trial managers. In response, a third, fully online version was developed and released in 2019, based on concepts developed and refined in the previous round of software design, but implemented anew. We describe the design, technology, and application of ClimMob version 3 in the following sections.

## 3 The structure of ClimMob

**Fig. 3:**
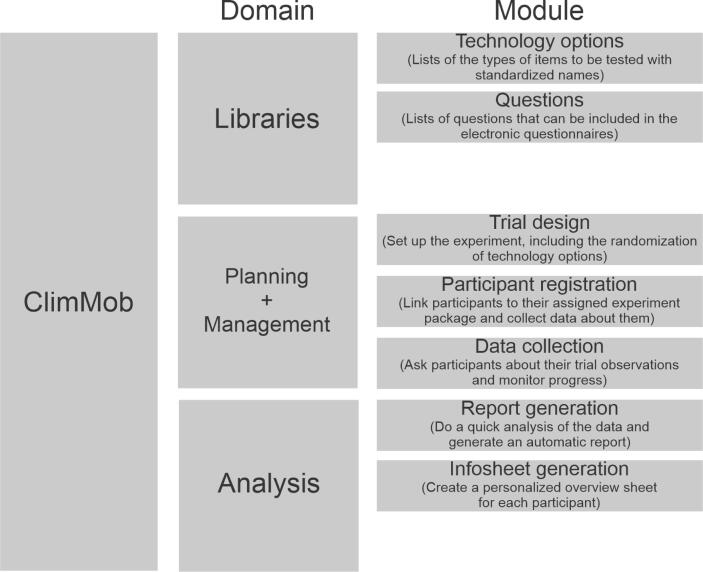
The conceptual structure of ClimMob.

ClimMob is divided into three domains: (1) Libraries, (2) Planning + Management, and (3) Analysis (Fig. 3). These domains interact with each other at different stages of the experimental cycle.

### 3.1 Libraries domain: Standardizing data collection

The libraries domain aims at standardizing the experimental treatments and the questions that will be used to ask in questionnaires presented to participants. The libraries are available to all users of the platform. The libraries domain is divided into two modules: Technology options and Questions. The following subsections discuss each.

#### 3.1.1 Technology options module

The technology options module contains standardized lists of technologies or experimental treatments. The Technologies module aims at facilitating ClimMob users to standardize the names of the technologies that are being evaluated in the field. This could be crop varieties, types of fertilizers, agronomic practices, or other treatments. Each treatment has different levels (or factors). For example, a *technology* could be “US heirloom varieties of maize” with *technology options*, “Lancaster Surecrop”, “Reid's Yellow Dent Corn”, etc. Technologies can be system-wide or user-defined. System-wide technologies and their options can be derived from standardized terms from external sources, such as Crop Ontology (Shrestha et al., 2012). A user-defined treatment, on the other hand, is initially only accessible to the user that created it. User-defined treatments are evaluated regularly to identify those that have the potential to become system-wide, facilitating a progressive standardization within the community of users.

#### 3.1.2 Questions module

The questions module aims at standardizing the questions used to evaluate treatments, to describe the trial conditions, and to characterize participants in the trials. The module allows users to create questions that capture different types of data, including numbers, text, GPS points, photos, and barcodes. To enhance standardization, the question types are based on Open Data Kit (ODK), an open initiative for collecting data on mobile devices (Hartung et al., 2010). ODK has a large user base and an active community of developers. It supports the creation of data collection forms. ClimMob creates ODK forms for all data collection (see 3.2.2, 3.2.3 below). In addition to the ODK question types, ClimMob adds a “triadic comparison” type which allows users to rank a set of technology options. This question type specifically supports the tricot approach (see Section 2 above).

Like the Technologies module, the Questions module can also be system-wide or user-defined. Where this is possible, system-wide questions are linked to ontological terms. Importantly, triadic traits are linked to Crop Ontology (Arnaud et al., 2020). User-defined questions are also evaluated regularly to identify those that have the potential to become system-wide thus also increasing the level of standardization within the community of users. This is an ongoing curation process that will be refined and progressively linked to ontologies over time.

### 3.2 Planning + Management domain: Supporting high-quality implementation

The Planning + Management domain supports the process of planning and managing a tricot trial across the experimental cycle. This domain is separated into three modules: Trial design, Participant registration, and Data collection.

#### 3.2.1 Trial design module

The Trial design module allows the user to structure a tricot trial. Most design choices involve including standardized information from the Libraries domain. The design process starts by defining the number of participants taking part in the trial and generic information like geographical coverage, contact information, and other metadata related to the trial. Afterwards, the user selects the Technologies (a category of options, e.g. “Maize varieties”) and Technology options (the different options within that category) from the Library that will be evaluated as part of the experiment. Finally, the user designs a questionnaire that will be used in the registration of participants with questions pulled from the Library and allocating them to user-defined sections.

ClimMob provides functionality for an incomplete block design (incomplete blocks of three items with items not repeated within blocks) considering the block size, the number of treatment levels, and the number of participants. The underlying code for the randomization is implemented in R in the package ClimMobTools (de Sousa et al., 2022b).

The experimental design addresses two challenges that are important to tricot experimentation. First, the trial should be robust to the loss of some of the plots, as some participants may fail to conduct observations or report results. The risk is that as a result of such losses, some technology options are compared only with a narrow set of other options. Secondly, the experimental design should balance the technology options across the area in which the experiment is conducted. For a trial involving different crop varieties, this means that all varieties should occur evenly in all the different villages.

To ensure robustness against data loss, the experimental design is approximately A-optimal. This type of optimality is the most robust against randomly missing blocks. It minimizes the effect that data loss can have on how technology options are compared to each other. Technically, this is assessed by constructing a graph that connects the technology options tested. Each time technology options are compared with each other in a tricot block of three, they have a connection. A-optimality is achieved by minimizing the Kirchhoff index of this graph, an index that indicates its connectivity (Bailey et al., 2009).

The experimental design is also “sequentially balanced”. This means that sets of consecutive blocks are near-optimally balanced. It also means that technology options will be balanced across geographical space, as long as packages with consecutive numbers are handed out in each village. For a consumer-testing exercise, handing out consecutive packages will achieve balance over time during the day. The sequential balancing also makes it easy to meet other balancing requirements (by type of farm, gender, etc.), if this is needed. Again, this can be done by assigning consecutive packages to each participant category. The technology options are also balanced across the positions in each incomplete block. In other words, each option occurs with the same frequency as first, second, or third. See Table 1 for an example.

**Table 1:** Example of a tricot experimental design for a group of 20 farmers: a sequentially balanced incomplete block design with 10 varieties (technology options) and 3 technology options per block (package).

Package	Option A	Option B	Option C
Package 1	INTA Matagalpa	INTA Ferroso	INTA Chinandega
Package 2	INTA Centro Sur	INTA Sur	INTA Ferroso Sequía
Package 3	INTA Fuerte Sequía	INTA Sequía	INTA Negro
Package 4	INTA Rojo	INTA Chinandega	INTA Centro Sur
Package 5	INTA Sequía	INTA Ferroso Sequía	INTA Rojo
Package 6	INTA Ferroso	INTA Matagalpa	INTA Sur
Package 7	INTA Negro	INTA Centro Sur	INTA Fuerte Sequía
Package 8	INTA Fuerte Sequía	INTA Rojo	INTA Ferroso
Package 9	INTA Chinandega	INTA Negro	INTA Matagalpa
Package 10	INTA Ferroso Sequía	INTA Chinandega	INTA Sequía
Package 11	INTA Sur	INTA Fuerte Sequía	INTA Ferroso Sequía
Package 12	INTA Matagalpa	INTA Centro Sur	INTA Sequía
Package 13	INTA Ferroso	INTA Sur	INTA Negro
Package 14	INTA Rojo	INTA Fuerte Sequía	INTA Matagalpa
Package 15	INTA Centro Sur	INTA Sequía	INTA Ferroso
Package 16	INTA Sur	INTA Negro	INTA Chinandega
Package 17	INTA Ferroso Sequía	INTA Ferroso	INTA Fuerte Sequía
Package 18	INTA Rojo	INTA Matagalpa	INTA Centro Sur
Package 19	INTA Sequía	INTA Rojo	INTA Sur
Package 20	INTA Chinandega	INTA Ferroso Sequía	INTA Negro

#### 3.2.2 Participant registration module

**Fig. 4:**
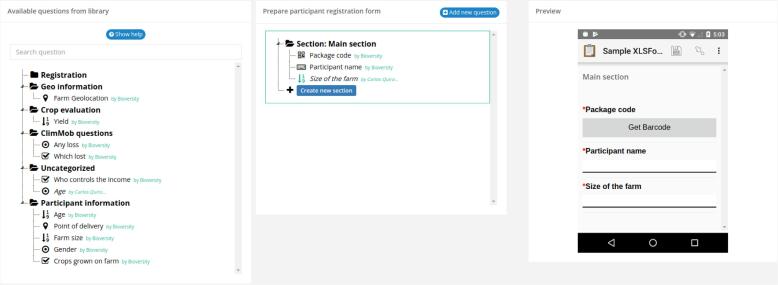
The ClimMob interface for building a registration form. The left part contains a ‘library’ with questions. These can be dragged to the middle part to include them in the questionnaire. The right part visualizes immediately how the questionnaire would look in the mobile app ODK Collect.

The registration of participants pulls question types from the Questions Module and allows the user to design one ODK data collection form to obtain information about each participant. During the registration of participants, it is of paramount importance to link each participant with the individual package of technology options they receive. To support this process, ClimMob has a form builder (see Fig. 4) to create a registration form. This form builder is WYSIWYG (What You See Is What You Get), which means that the right part of the interface shows immediately how the electronic questionnaire would look like in the mobile app ODK Collect.

Field agents register participants using the ODK Collect app. They link each participant to a trial package by scanning its QR code. These QR codes are generated automatically by ClimMob for package preparation. They need to be printed and attached to each package during package preparation. Using QR codes reduces mistakes during registration. The general dashboard in ClimMob will show the progress of different field agents in participant registration.

#### 3.2.3 Data collection module

**Fig. 5:**
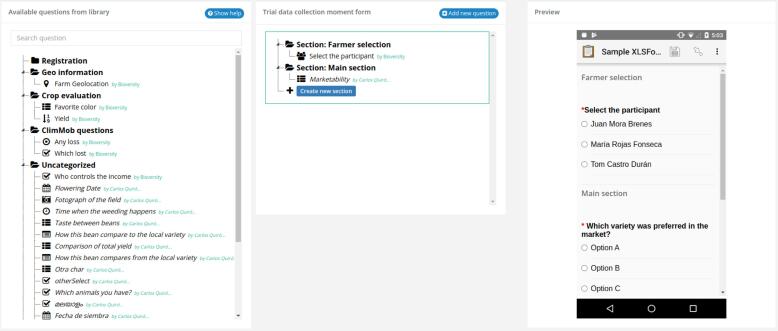
The ClimMob interface for building a data collection form. Its functionality is similar to that for the registration form (Fig. 4).

The Data collection module pulls question types from the Questions module and allows the user to design any number of ODK data collection forms to evaluate the performance of a tricot trial. Within a tricot trial, data collection can happen at different “data collection moments”, for example, “at flowering stage” or “after harvest”. Each data collection moment requires a designated data collection form. During participants’ registration, it is important to link the progress information with each participant providing it. To support this, each data collection form has an ODK search and select widget to identify and pick each participant. See Fig. 5.

During each data collection moment, technology options can be ranked in a triadic comparison, or participants can be asked questions that serve as explanatory variables in the analysis. The latter questions relate to possible factors that influence crop performance or evaluation, for example, whether a certain crop management practice was performed.

Each data collection is performed by field agents using the ODK Collect App. The general dashboard in ClimMob will show the progress of different field agents in each data collection.

### 3.3 Analysis domain: Supporting fast feedback

**Fig. 6:**
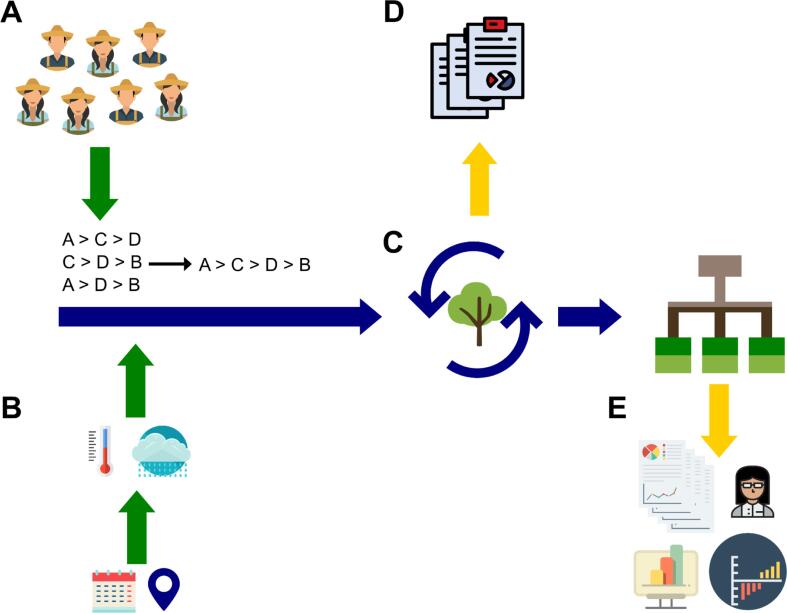
Analytical workflow implemented on ClimMob for the production of automated reports. (A) Multiple participants contribute with small tasks. Ranking-based approaches implemented in the packages *gosset* and *ClimMobTools* allow the data to be easily aggregated. (B) Explanatory agroclimatic covariates are added using geographic coordinates and planting dates with the packages *climatrends* and *chirps*. (C) Rankings are pushed to the model fitting module implemented using *PlackettLuce* and *gosset* packages. A forward selection approach is used to find the best model. (D) Automated reports and feedback to participants in (A) are generated. (E) Supervised analysis combining ClimMob projects is often done locally to test new hypotheses, try new analytical implementations and produce scientific papers. New workflows, analytical and visualization approaches validated in (E) are often generalized and integrated to (C) to enrich the automated reports.

The objective of the Analysis domain is to provide quick feedback on the performance of the technologies being tested in a given ClimMob project. Data standardization facilitates the generation of standard information outputs, including overview reports for trial managers and personalized result sheets for participants, for each project designed and managed on ClimMob (Fig. 6).

The analysis workflow is implemented as a backend process on ClimMob, it involves a series of R packages (See Table 2) developed to fetch, organize and analyze the data produced in the ClimMob projects (de Sousa et al., 2022a). The packages are targeted to function at a ClimMob workflow but their majority are also able to operate in other domains outside the platform. The workflow is implemented in a modular approach which allows producing different reports for different audiences (e.g. project managers, stakeholders, and participants). The package *ClimMobTools* (de Sousa et al., 2022b) connects to ClimMob via API to allow the user to import tricot data into the R environment. Additionally the package offers a data wrangling function to transform the raw tricot ranking data into a Plackett-Luce ranking for the data analysis. The *PlackettLuce* package (Turner et al., 2020) implements a generalization of the model independently proposed by Luce (1959) and Plackett (1975) for modeling rankings data. The model estimates the probability of one item (a given technology in a ClimMob project) to outperform other items in a set. It is the core R package for data analysis in ClimMob. The R package *gosset* (de Sousa et al., 2022c) provides supporting tools for data analysis and visualization. ClimMob projects with a focus on agriculture and crop ecology can take advantage of R package *climatrends* (de Sousa et al., 2023) which provide methods and tools to compute agro-climatic indices that can be linked to the ClimMob data for analysis using Plackett-Luce trees. The climatic data required to compute such indices can be provided by the user (e.g. from data loggers) or alternatively using the R packages *nasapower* (Sparks, 2018) or *chirps* (de Sousa et al., 2020).

**Table 2:** R packages generated as a result of research on tricot data which are currently used in ClimMob.

Package	Description	Webpage	Reference
	API client for the ClimMob platform. Project managers can access their project data in R and run deeper analysis.	https://CRAN.R-project.org/package=ClimMobTools	De Sousa et al., (2021a)
	Tools to handle and visualize the tricot rankings.	https://CRAN.R-project.org/package=gosset	De Sousa et al., (2022b)
	Tools and methods to compute environmental indices. Project managers can compute the agro-climatic indices using their own set of climate data or alternatively the NASA POWER data provided via the R package nasapower (Sparks, 2018)	https://CRAN.R-project.org/package=climatrends	De Sousa et al. (2023)
	API client for the CHIRPS and CHIRTS databases with daily rainfall and temperature data.	https://CRAN.R-project.org/package=chirps	De Sousa et al. (2020a)
	Implements the Plackett-Luce model in R	https://CRAN.R-project.org/package=PlackettLuce	Turner et al. (2020)

The ClimMob analytical workflow (Fig. 6) is implemented in R and executed on ClimMob via its web interface (see Section 3.3 above). The user selects the parameters and elements that should be analyzed for the report, including the type(s) of report that should be generated. The basic stack communicates with the analytical workflow sending the parameters as command lines and the data as a JSON file. The analytical workflow parses the data, runs the analysis, and produces the report which is sent to the Storage part. ClimMob generates a link to download the report and the additional outputs in the Download section on the Web Interface. This process takes between 3 and 7 min to complete, depending on the volume of data to be parsed and analyzed. The process is limited to individual ClimMob projects. Users with programming skills can read data from different ClimMob projects into R, and do a deeper analysis of the data using different R packages (de Sousa et al., 2022a).

## 4 Architectural design

Design criteria for the architecture of ClimMob were: (1) that it should implement Component-Based Software Engineering (CBSE), to allow for a flexible, modular design of software that is easy to maintain, (2) that code should be open-source, and (3) that different components should have a broad user community, to ensure their continuity in the future.

The architecture of ClimMob is inspired by CKAN (https://ckan.org/), one of the most widely used data catalog systems. CKAN is written in the Python programming language. It uses Pyramid, a Python-based web framework, and Jinja2, a templating engine. CKAŃs component architecture is based on PyUtilib Component Architecture (PCA), which follows CBSE principles (Hart and Siirola, 2010). New frontend and backend capabilities can be easily added through extensions, which connect with the CKAN core package through one or more plugins. We decided to use this same combination of technologies for the basic stack of ClimMob.

For data collection, we selected Open Data Kit (ODK). This is a robust open-source app that has been developed for field data in the Global South (Hartung et al., 2010). It enables data collection in a wide range of formats in terms of the types of questions and input formats (including GPS points, audio, and image). Also, it has an extensive user base, which also benefits ClimMob users.

An important limitation of ODK is that it produces data in XML format, but to make data available for easy future use and allow for aggregated analysis, it needs to be stored in a relational way. Therefore, we decided to transform user-defined ODK surveys into MySQL schemata and store submissions as relational data. To do this, we use ODK Tools, which is the only available open source toolbox with this functionality (Quiros, 2022a, Quiros, 2022b).

**Fig. 7:**
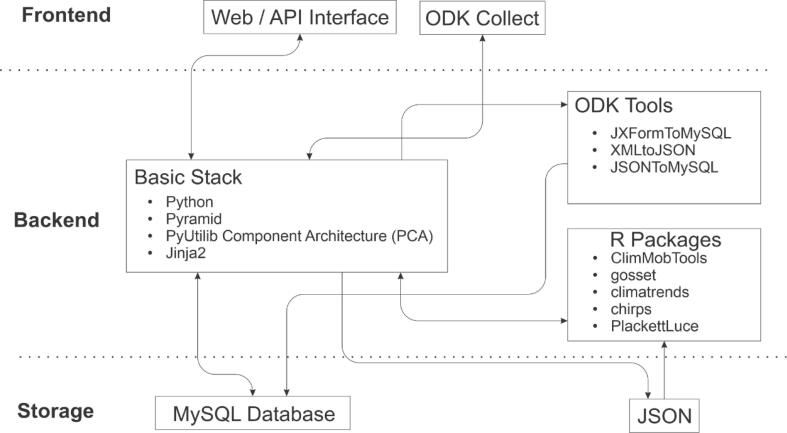
The architectural design of ClimMob.

In the following sections, we describe ClimMob́s architecture in more detail. It is divided into three parts: storage, backend, and frontend (Fig. 7).

### 4.1 Frontend

The users of ClimMob control all aspects of the trial design and data analysis through the web Interface. This interface combines open-source technologies and languages including JQuery and CSS. Developers and other technical users can use the API to interface ClimMob with other third-party applications. Field agents collect data using ODK Collect. The web interface, API interface and ODK Collect interact all three with the backend of ClimMob through HTTP requests.

### 4.2 Backend

The backend of ClimMob consists of three main components: 1) the basic stack, 2) ODK Tools, and 3) the analytical workflow. We discuss each in turn below.

#### 4.2.1 The basic stack

The basic stack is entirely constructed with Python. The basic stack receives user requests from the web or API interface through a web application built with Pyramid. Requests from the web browser are processed by the Pyramid web application and results are passed to Jinja2, which renders the HTML along with all the necessary JavaScript and CSS components before returning the content to the user. Responding to API requests, the web application returns a JSON object.

The basic stack implements PCA to define extension points across the application. PCA allows to extend or modify the internal processes of ClimMob through plug-able components without the need of modifying the main source code. For example, a new plug-able component could be added to send end-of-day progress messages to field assistants over WhatsApp.

The basic stack creates ODK data collection forms and serves them through ODK Collect, an Android app. The basic stack receives each ODK submission, delegating the processing of the submission to ODK Tools (see next subsection).

#### 4.2.2 ODK Tools

**Fig. 8:**
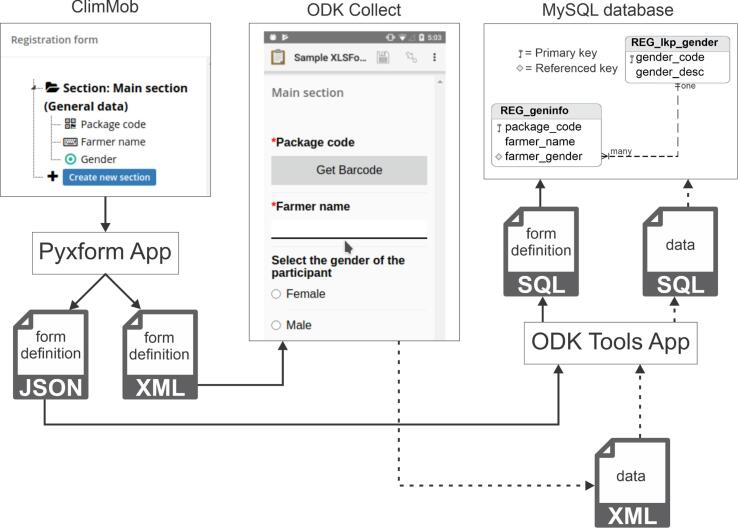
Transformation of a form from the ClimMob’s form designer to ODK Collect and MySQL schema. The dotted line indicates data flow from ODK Collect to MySQL.

ODK Tools is an open-source toolbox for processing ODK submissions into MySQL tables. ClimMob allows the user to create any ODK form to register participants into a study and to follow up on the performance of technology options within a tricot trial. Each ODK form created by ClimMob is firstly saved as an ODK Excel file, which is then rendered by PyXForm as JSON and XML. The JSON output of PyXForm is processed by ODK Tools to generate a MySQL schema to store the submissions (see Fig. 8). Each submission arrives first at ClimMob in XML format and then gets inserted into MySQL by ODK Tools.

### 4.3 Data storage and analytics

**Fig. 9:**
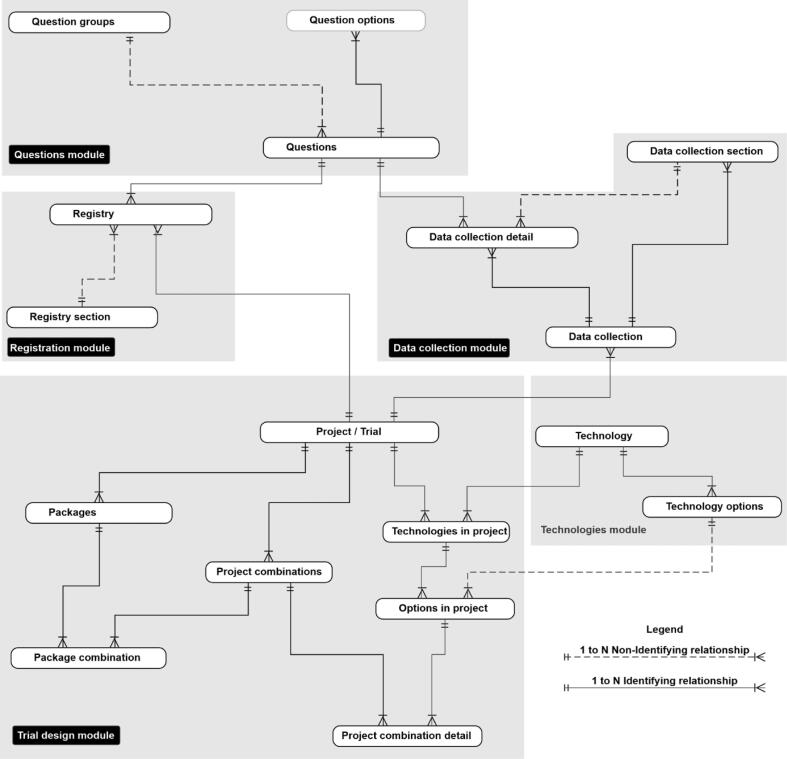
Most important tables that control the flow, structure, and features of ClimMob.

ClimMob stores all data in a MySQL database. The data for the ClimMob platform is stored across 26 tables to control the flow, structure, and features of ClimMob. Fig. 9 shows the most important tables and the modules that they belong to. The appendix has a detailed description of all tables.

The ClimMob platform relies on the R package ClimMobTools to make the data available for scientific analysis. ClimMob takes the trial data from the MySQL database, turns it into a JSON file, and makes it available to ClimMobTools, which reads them into the R environment. This internal process is equivalent to the process available for users who use ClimMobTools in their own R environment and access the data through API. Within R, a script is run to analyze the data and produce a report, containing text, tables and figures. This script uses several of the R packages presented in Table 2.

## 5 User reception

**Fig. 10:**
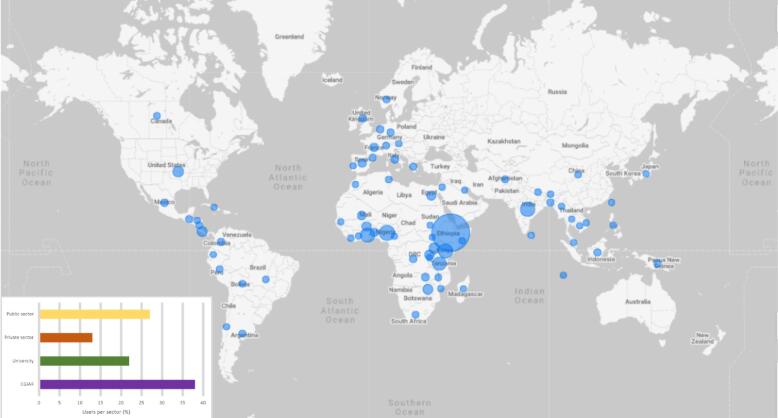
Users of ClimMob by geographic location and their distribution across sectors. The size of the bubble represents the number of users at a location in relation to other locations.

Currently, the ClimMob platform has close to 800 users, most of them from the Global South (see Fig. 10). The single largest group of users belongs to CGIAR, a consortium of international agricultural research institutes. However, users from other public organizations and universities constitute the majority of ClimMob users, when taken together. The private sector, including seed and input companies, though currently a small group of users, is important for the financial sustainability of ClimMob.

In the first quarter of 2022, the development team asked the community of users to evaluate their perception of ClimMob using two different metrics. Sixteen users were asked to answer ten questions to obtain the System Usability Scale (SUS), and 25 users were requested to answer two questions to obtain the UMUX-LITE, which is based on the Usability Metric for User Experience (UMUX) (Lewis et al., 2013). ClimMob received a SUS of 68.1 (95 % confidence interval: 5.1)) and a UMUX Lite score of 73.0 (95 % confidence interval: 6.8), which corresponds to ∼70 on the SUS scale (Borsci et al., 2015). This sample size is larger than the recommended minimum sample size of 10 testers (Sauro 2010, p. 51). These scores are consistent, have relatively small confidence intervals, and are close to the median score for software usability (a SUS of 68). This means that ClimMob is better than 50 % of a large reference set of software (Brooke, 2013). In terms of a descriptive adjective, the scores imply that ClimMob’s usability is closest to “good”.

## 6 Planned features

The current development activities on ClimMob focuses on improving the tools for future use in four main areas: a) enhanced user experience, b) improved data collection, c) increased integration and interaction with other systems, and d) improved ClimMob reports to better inform crop improvement decision-makers.

### 6.1 Enhanced user experience

Through continuous efforts in user training and support, we collect feedback from users on how to improve the overall experience of the platform. These new features will allow us to maintain or improve our SUS score in the future. This work will continue in the 1000FARMS project, a 4-year project to mainstream the tricot approach in crop breeding (https://1000farms.net/). This project will allow us to increase interaction with national breeding programs and other actors to further enhance user experience.

### 6.2 Improved data collection

Though most of the users of ClimMob build data collection surveys using the web interface, many advanced users would like to create their own ODK surveys designed in Excel. In this way, users can use the full functionality of ODK, including skip logic, which can send respondents to a different question in the survey based on their response. For example, users may want to capture household information from tricot participants using the Rural Household Multiple Indicator Survey (RHoMIS) (Hammond et al., 2017). We plan to implement the possibility to use Excel to flexibly design ODK surveys for ClimMob by providing integration between ClimMob and FormShare. The latter is a comprehensive platform for ODK surveys (Quiros, 2022a, Quiros, 2022b).

ClimMob collects data using ODK. This works well for data collection with field agents with Android phones, who have access to the Internet at least periodically (not necessarily in the field, as data can be uploaded later). For other situations, we plan to add other channels for receiving information like USSD, IVR, and SMS that would allow farmers in very remote locations to participate in on-farm trials.

### 6.3 Increased integration and interaction with other systems

Crop breeders will need a seamless flow of data between ClimMob and the digital breeding platforms that they currently use to make decisions using the data produced by tricot trials, and to integrate it with other types of data, such as genomic data (for example, de Sousa et al., 2021a, de Sousa et al., 2021b). Therefore, integration and interaction with other breeding platforms are key to the success of ClimMob. Through 1000FARMS, a 4-year project funded by Bill and Melinda Gates Foundation, we will integrate ClimMob with other digital platforms such as the Enterprise Breeding System (EBS).

### 6.4 Improved ClimMob reports to better inform crop improvement decision-makers

At present, ClimMob produces a general report for decision-makers. However, different types of decision-makers use this information for different types of decisions. For example, a breeding programme uses the data to choose the candidate variety for variety release, and subsequently a variety release committee decides to release the variety officially for commercial use. These two groups of users need different types of reports. We will design a more differentiated set of reports tailored to different user types, through a user-centered design process. The majority of the ClimMob users work for the public or NGO sectors, and fewer users are in the private sector, including seed companies, farmer organizations, and farm service providers. We will address the full range of user types in this process.

## 7 Final reflections

The design process leading to the current version of ClimMob has provided important lessons. First of all, the simultaneous design of a workflow and a digital platform, the iterative improvement approach, and the reception of constant user feedback, has been important for growing a user base. Working with a small, dedicated team has made it possible to manage complexity while keeping a coherent conceptual vision. Establishing the basic methodological concepts for the workflow early through prototyping brought focus to the development process and has made it possible to show the viability of digital transformation of a field of practice. This contrasts with fitting digital tools to an extant workflow and enhancing it incrementally.

To obtain constant user feedback, it has been important to keep communication lines short. Scientists and developers co-taught courses on implementing the tricot approach and using the ClimMob software. These were unique occasions, in which they jointly observed and discussed many unanticipated obstacles and opportunities. This include how users interact with the interface, practical challenges in the field, and options to collect complementary information from farmers.

In terms of architectural design, our decision to re-program ClimMob using a component architecture like PCA has yielded many benefits. The private sector, for example, can create value-added functionality and make a profit without modifying the original source code. In the same way, custom versions of ClimMob can be tailored to the specific needs of projects and programs, this is for example the case of 1000FARMS (https://1000farms.climmob.net/). Moving the data collection to ODK and processing submissions with ODK Tools was also a very strategic move that allow users to collect any kind of data while storing it relationally and therefore be able to run diverse analytics across projects.

Lastly, it is important that ClimMob is made available as open-source software. This makes it possible to shape scientific collaborations to improve the software further, while shaping entrepreneurial efforts that make ClimMob and associated services widely available, and to facilitate scaling its use through a digital ecosystem business model. We are already offering commercial services with ClimMob, while maintaining a free version for use by public institutions in the Global South. We believe that this can attract talented young entrepreneurs to further tailor ClimMob, repurpose different components, and contribute with their entrepreneurial efforts to maintain a viable digital ecosystem, while delivering useful digital services that facilitate user-centric innovation.

## CRediT authorship contribution statement

**Carlos Quirós:** Investigation, Conceptualization, Supervision, Validation, Software, Writing – original draft, Writing – review & editing. **Kauê de Sousa:** Conceptualization, Investigation, Methodology, Writing – original draft, Software. **Jonathan Steinke:** Investigation, Writing – original draft. **Brandon Madriz:** Investigation, Software, Writing – original draft. **Marie-Angélique Laporte** Investigation, Writing – original draft. **Elizabeth Arnaud:** Investigation, Writing – original draft. **Rhys Manners:** Investigation, Writing – original draft. **Berta Ortiz-Crespo:** Investigation, Writing – original draft. **Anna Müller:** Investigation, Writing – original draft. **Jacob van Etten:** Conceptualization, Investigation, Methodology, Project administration, Supervision, Validation, Writing – original draft, Writing – review & editing, Software.

## Declaration of competing interest

The authors declare that they have no known competing financial interests or personal relationships that could have appeared to influence the work reported in this paper.

## Data Availability

Data will be made available on request.
