# Supplementary material for: ClimMob: Software to support experimental citizen science in agriculture
Source: Comput Electron Agric. 2024 Feb;217:None. doi: 10.1016/j.compag.2023.108539 (PMC10853689; doi:10.1016/j.compag.2023.108539)
Supplement: Supplementary data 1 [file mmc1.docx]

Appendix: The tables that contain the data and metadata on the ClimMob platform

| **Table** |  | |  |  |
| --- | --- | --- | --- | --- |
| **assdetail** | *Detail of a data collection moment* | |  |  |
|  | **Field** | **Description** | **Type** | **Key** |
|  | project_id | Project ID | varchar(64) | X |
|  | ass_cod | Data collection ID | varchar(80) | X |
|  | question_id | Question ID | int | X |
|  | section_project_id | Section – Project ID | varchar(80) |  |
|  | section_assessment | Section – Data collection ID | varchar(80) |  |
|  | section_id | Section ID | int |  |
|  | question_order | Question order | int |  |
|  |  |  |  |  |
| **assessment** | *Data collection moment* | |  |  |
|  | **Field** | **Description** | **Type** | **Key** |
|  | project_id | Project ID | varchar(64) | X |
|  | ass_cod | Data collection ID | varchar(80) | X |
|  | ass_desc | Data collection description | varchar(120) |  |
|  | ass_days | Start at number of days from planting | int |  |
|  | ass_status | Data collection status | int |  |
|  | ass_final | Whether the data collection is the final | int |  |
|  | extra | Extra data in JSON format | mediumtext |  |
|  |  |  |  |  |
| **asssection** | *Section of a data collection moment* | |  |  |
|  | **Field** | **Description** | **Type** | **Key** |
|  | project_id | Project ID | varchar(64) | X |
|  | ass_cod | Data collection ID | varchar(80) | X |
|  | section_id | Section ID | int | X |
|  | section_name | Section name | varchar(120) |  |
|  | section_content | Section content | mediumtext |  |
|  | section_order | Section order | int |  |
|  | section_private | Whether the section is private | int |  |
|  |  |  |  |  |
| **country** | *Countries* | |  |  |
|  | **Field** | **Description** | **Type** | **Key** |
|  | cnty_cod | Country code | varchar(3) | X |
|  | cnty_name | Country name | varchar(120) |  |
|  | cnty_iso | Country ISO code | varchar(3) |  |
|  |  |  |  |  |
| **crop** | *Crops* | |  |  |
|  | **Field** | **Description** | **Type** | **Key** |
|  | crop_code | Crop code | int | X |
|  | crop_name | Crop description | varchar(45) |  |
|  |  |  |  |  |
| **enumerator** | *Field agents* | |  |  |
|  | **Field** | **Description** | **Type** | **Key** |
|  | user_name | User ID | varchar(80) | X |
|  | enum_id | Field agent ID | varchar(80) | X |
|  | enum_name | Field agent name | varchar(120) |  |
|  | enum_password | Field agent password (encrypted) | mediumtext |  |
|  | enum_active | Whether the field agent is active | tinyint |  |
|  | enum_telephone | Field agent telephone | varchar(120) |  |
|  | enum_email | Field agent email | varchar(120) |  |
|  | extra | Extra data in JSON format | mediumtext |  |
|  |  |  |  |  |
| **package** | *Project / trial packages* | |  |  |
|  |  |  |  |  |
|  | **Field** | **Description** | **Type** | **Key** |
|  | project_id | Project ID | varchar(64) | X |
|  | package_id | Package ID | int | X |
|  | package_code | Package Code | varchar(45) |  |
|  | package_image | Package QR image | blob |  |
|  |  |  |  |  |
| **pkgcomb** | *Combination of technology options in a package* | |  |  |
|  | **Field** | **Description** | **Type** | **Key** |
|  | project_id | Project ID | varchar(64) | X |
|  | package_id | Package ID | int | X |
|  | comb_project_id | Combination – Project ID | varchar(64) | X |
|  | comb_code | Combination code | int | X |
|  | comb_order | Combination order | int |  |
|  |  |  |  |  |
| **prjalias** | *Technology options used in project / trial* | |  |  |
|  | **Field** | **Description** | **Type** | **Key** |
|  | project_id | Project ID | varchar(64) | X |
|  | tech_id | Technology ID | int | X |
|  | alias_id | Technology option ID | int | X |
|  | tech_used | Technology used from library | int |  |
|  | alias_used | Technology options used from library | int |  |
|  | alias_name | User defined technology option (if not in library) | varchar(120) |  |
|  |  |  |  |  |
| **prjcnty** | *Countries where the trial happens* | |  |  |
|  | **Field** | **Description** | **Type** | **Key** |
|  | project_id | Project ID | varchar(64) | X |
|  | cnty_cod | Country code | varchar(3) | X |
|  | cnty_contact | Contact in the country for the trial | varchar(120) |  |
|  |  |  |  |  |
| **prjcombdet** | *Detail of a combination in a project* | |  |  |
|  | **Field** | **Description** | **Type** | **Key** |
|  | project_id | Project ID | varchar(64) | X |
|  | comb_code | Combination code | int | X |
|  | project_id_tech | Options in project – Project ID | varchar(64) | X |
|  | tech_id | Options in project – Technology ID | int | X |
|  | alias_id | Options in project – Technology option ID | int | X |
|  | alias_order | Technology option order | int |  |
|  |  |  |  |  |
| **prjcombination** | *Combination of technology options available in a project* | |  |  |
|  | **Field** | **Description** | **Type** | **Key** |
|  | project_id | Project ID | varchar(64) | X |
|  | comb_code | Combination code | int | X |
|  | comb_usable | Whether the combination is usable | tinyint |  |
|  | quantity_available | Quantity of seed available for this combination | int |  |
|  |  |  |  |  |
| **prjenumerator** | *Field agents in a project / trial* | |  |  |
|  | **Field** | **Description** | **Type** | **Key** |
|  | project_id | Project ID | varchar(64) | PRI |
|  | enum_user | User ID | varchar(80) | PRI |
|  | enum_id | Field agent ID | varchar(80) | PRI |
|  | extra | Extra data in JSON format | mediumtext |  |
|  |  |  |  |  |
| **prjtech** | *Technology used in project / trial* | |  |  |
|  | **Field** | **Description** | **Type** | **Key** |
|  | project_id | Project ID | varchar(64) | X |
|  | tech_id | Technology ID | int | X |
|  |  |  |  |  |
| **products** | *Products generates in a project / trial* | |  |  |
|  | **Field** | **Description** | **Type** | **Key** |
|  | celery_taskid | Celery Task ID | varchar(80) | X |
|  | product_id | Product ID | varchar(80) | X |
|  | datetime_added | Date and time created | datetime |  |
|  | output_id | Output ID | varchar(80) |  |
|  | output_mimetype | Output mimetype | varchar(80) |  |
|  | process_name | Process name | varchar(80) |  |
|  | project_id | Project ID that the project belongs to | varchar(64) |  |
|  |  |  |  |  |
| **project** | *Project / trial definition* | |  |  |
|  | **Field** | **Description** | **Type** | **Key** |
|  | project_id | Project ID | varchar(64) | X |
|  | project_cod | Project code | varchar(80) |  |
|  | project_name | Project name | varchar(120) |  |
|  | project_abstract | Project abstract | mediumtext |  |
|  | project_tags | Project tags | mediumtext |  |
|  | project_pi | Project PI | varchar(120) |  |
|  | project_piemail | Email of the project PI | varchar(120) |  |
|  | project_active | Whether the project is active | tinyint |  |
|  | project_public | Whether the project is public | tinyint |  |
|  | project_numobs | Number of participants in the trial | int |  |
|  | project_numcom | Number of options to evaluate (e.g., 3 = tricot) | int |  |
|  | project_lat | Project latitude | varchar(120) |  |
|  | project_lon | Project longitude | varchar(120) |  |
|  | project_creationdate | Creation date | datetime |  |
|  | extra | Extra data in JSON format | mediumtext |  |
|  | project_assstatus | Whether the project’s data collection has started | int |  |
|  | project_regstatus | Whether the project’s registration has started | int |  |
|  | project_createcomb | Whether the project has combination | int |  |
|  | project_createpkgs | Whether the project has packages | int |  |
|  | project_localvariety | Whether the trial compares against a local variety | int |  |
|  | project_cnty | Country where the trial happens | varchar(3) |  |
|  | project_registration_and_analysis | Whether the project’s has data collection moments | int |  |
|  | project_label_a | Default label for option A (A by default) | varchar(120) |  |
|  | project_label_b | Default label for option B (B by default) | varchar(120) |  |
|  | project_label_c | Default label for option C (C by default) | varchar(120) |  |
|  | project_template | Whether the project is template | int |  |
|  |  |  |  |  |
| **qstgroups** | *Question groups* | |  |  |
|  | **Field** | **Description** | **Type** | **Key** |
|  | user_name | User ID | varchar(80) | X |
|  | qstgroups_id | Question group ID | varchar(80) | X |
|  | qstgroups_name | Question group name | varchar(120) |  |
|  |  |  |  |  |
| **qstoption** | *Option values of a single or multiple select question* | |  |  |
|  | **Field** | **Description** | **Type** | **Key** |
|  | question_id | Question ID | int | X |
|  | value_code | Value code | varchar(80) | X |
|  | value_desc | Value description | varchar(120) |  |
|  | value_isother | Whether the value is other | int |  |
|  | value_isna | Whether the value is NA | int |  |
|  | value_order | Order of the value | int |  |
|  |  |  |  |  |
| **question** | *Library of questions* | |  |  |
|  | **Field** | **Description** | **Type** | **Key** |
|  | question_id | Question ID | int | X |
|  | question_desc | Question description | varchar(120) |  |
|  | question_notes | Long description | mediumtext |  |
|  | question_unit | Unit | varchar(120) |  |
|  | question_dtype | Question type | int |  |
|  | question_reqinreg | Whether the question is required in the registration of participants | tinyint |  |
|  | question_reqinasses | Whether the question is required in data collection moments | tinyint |  |
|  | user_name | User ID that this questions belongs to | varchar(80) |  |
|  | question_posstm | First question asked | varchar(120) |  |
|  | question_negstm | Second questions asked | varchar(120) |  |
|  | question_requiredvalue | Whether the question needs to be answered | int |  |
|  | extra | Extra data in JSON format | mediumtext |  |
|  | question_asskey | Whether the question is key in data collections | int |  |
|  | question_code | Question code | varchar(120) |  |
|  | question_regkey | Whether the question is key in the registration of participants | int |  |
|  | question_overall | Whether the question is the overall ranking | int |  |
|  | question_perfstmt | Performance question asked | varchar(120) |  |
|  | question_alwaysinasse | Whether the question should appear in data collection moments | int |  |
|  | question_alwaysinreg | Whether the question should appear in the registration of participants | int |  |
|  | question_fname | Whether the question is the participant name | int |  |
|  | question_overallperf | Whether the question is the overall performance | int |  |
|  | qstgroups_user | Question group – User name | varchar(80) |  |
|  | qstgroups_id | Question group – Group ID | varchar(80) |  |
|  | question_visible | Whether the question is visible | int |  |
|  | question_name | Question description in ODK Collect | varchar(120) |  |
|  | question_notobserved | Whether the question allows not observed | int |  |
|  | question_tied | Whether the question allows tied | int |  |
|  | question_quantitative | Whether the question is a quantitative value | int |  |
|  | question_forms | Whether the question is allows to appear in the registration or in a data collection | int |  |
|  |  |  |  |  |
| **registry** | *Questions in the registration of participants* | |  |  |
|  | **Field** | **Description** | **Type** | **Key** |
|  | project_id | Project ID | varchar(64) | X |
|  | question_id | Question ID | int | X |
|  | section_project_id | Registry Section – Project ID | varchar(64) |  |
|  | section_id | Registry Section – Section ID | int |  |
|  | question_order | Order of the questions in the registry | int |  |
|  |  |  |  |  |
| **regsection** | *Sections in the registration of participants* | |  |  |
|  | **Field** | **Description** | **Type** | **Key** |
|  | project_id | Project ID | varchar(64) | PRI |
|  | section_id | Section ID | int | PRI |
|  | section_name | Section name | varchar(45) |  |
|  | section_content | Section content | mediumtext |  |
|  | section_order | Section order | int |  |
|  | section_private | Whether the section is private | int |  |
|  |  |  |  |  |
| **sector** | *Sectors that a user could belong to* | |  |  |
|  | **Field** | **Description** | **Type** | **Key** |
|  | sector_cod | Sector code | int | X |
|  | sector_name | Sector name | varchar(120) |  |
|  |  |  |  |  |
| **techalias** | *Library of technology options* | |  |  |
|  | **Field** | **Description** | **Type** | **Key** |
|  | tech_id | Technology ID | int | X |
|  | alias_id | Technology option ID | int | X |
|  | alias_name | Technology option name | varchar(120) |  |
|  |  |  |  |  |
| **technology** | *Library of technologies* | |  |  |
|  | **Field** | **Description** | **Type** | **Key** |
|  | tech_id | Technology ID | int | X |
|  | tech_name | Technology name | varchar(45) |  |
|  | user_name | User that owns the technology | varchar(80) |  |
|  | crop_code | Crop assigned to the technology | int |  |
|  |  |  |  |  |
| **user** | *The users of ClimMob* | |  |  |
|  | **Field** | **Description** | **Type** | **Key** |
|  | user_name | User ID | varchar(80) | X |
|  | user_fullname | User name | varchar(120) |  |
|  | user_joindate | Date when the user joined ClimMob | datetime |  |
|  | user_password | User password (encrypted) | mediumtext |  |
|  | user_organization | Organization of the user | varchar(120) |  |
|  | user_email | User email | varchar(120) |  |
|  | user_apikey | User API key | varchar(45) |  |
|  | user_about | Information about the user | mediumtext |  |
|  | user_cnty | Country of the user | varchar(3) |  |
|  | user_sector | Sector of the user | int |  |
|  | user_active | Whether the user is active or not | tinyint |  |
|  | extra | Extra data in JSON format | mediumtext |  |
|  |  |  |  |  |
| **user_project** | *Projects that a user has access to* | |  |  |
|  | **Field** | **Description** | **Type** | **Key** |
|  | project_id | Project ID | varchar(64) | X |
|  | user_name | User ID | varchar(80) | X |
|  | access_type | Access type | int |  |
|  | project_dashboard | Whether the project is active in the dashboard | int |  |
